# Through the Haze of Hemorrhage: Unraveling Leptospirosis With Diffuse Alveolar Hemorrhage

**DOI:** 10.7759/cureus.82006

**Published:** 2025-04-10

**Authors:** Mahavir Vasireddy, Nanthakumar Logithasan, Narayanasamy Senthil, Avinash Chenguttuvan, Prathyusha Gandikota

**Affiliations:** 1 General Internal Medicine, Sri Ramachandra Institute of Higher Education and Research, Chennai, IND; 2 General Medicine, Sri Ramachandra Institute of Higher Education and Research, Chennai, IND; 3 Internal Medicine, Sri Ramachandra Institute of Higher Education and Research, Chennai, IND

**Keywords:** diffuse alveolar hemorrhage, igm elisa, leptospirosis with severe clinical manifestation, microscopic agglutination test, tropical fever

## Abstract

Leptospirosis is a zoonotic infection caused by *Leptospira* species, presenting with a wide spectrum of clinical manifestations ranging from mild febrile illness to severe multi-organ dysfunction, also known as Weil’s disease. This case describes a patient with severe leptospirosis complicated by diffuse alveolar hemorrhage, acute kidney injury, hepatic dysfunction, and thrombocytopenia. The diagnosis was initially challenging due to negative serology in the early phase of the illness, but strong clinical suspicion led to a repeat *Leptospira* IgM positivity. Later, the diagnosis was confirmed with the microscopic agglutination test. The patient responded well to antibiotic therapy and supportive care, emphasizing the importance of clinical suspicion and repeat testing in severe cases of leptospirosis.

## Introduction

Leptospirosis is a globally significant zoonotic infection caused by the *Leptospira* species, a spirochete bacterium transmitted through direct or indirect contact with contaminated water, soil, or infected animal urine. The disease is particularly prevalent in tropical and subtropical regions, with an estimated one million cases and nearly 60,000 deaths annually worldwide [1]. In coastal India, leptospirosis is endemic, particularly during the monsoon season, with a pooled case fatality rate of 11% (95% confidence interval = 8-15%) reported in a recent systematic review of Indian studies [2].

Leptospirosis exhibits a wide spectrum of clinical manifestations, ranging from a mild febrile illness to severe multi-organ dysfunction, known as Weil’s disease [3]. Weil’s disease is characterized by hepatic dysfunction, acute kidney injury, thrombocytopenia, and pulmonary involvement [4]. Among its severe complications, leptospiral pulmonary hemorrhage syndrome (LPHS) is one of the most fatal, leading to diffuse alveolar hemorrhage (DAH) and acute respiratory failure, with mortality rates exceeding 50% in untreated cases [5].

Diagnosing leptospirosis remains challenging due to its nonspecific symptoms and overlap with other tropical febrile illnesses, such as dengue fever, malaria, scrub typhus, and viral hemorrhagic fevers [6]. Laboratory confirmation relies on serologic and molecular tests, with IgM enzyme-linked immunosorbent assay (ELISA) and microscopic agglutination test (MAT) being commonly used. However, IgM ELISA may be falsely negative in the early phase, necessitating repeat testing [7].

## Case presentation

A 19-year-old male presented with a history of high-grade fever for five days, associated with chills, rigors, headache, and multiple joint pains. There were no initial complaints of cough, shortness of breath, dysuria, abdominal pain, or vomiting. On day five of illness, the patient developed hypotension and was taken to an outside hospital, where he was given fluid resuscitation with normal saline and started on noradrenaline. Laboratory investigations at that time revealed thrombocytopenia (platelet count of 70,000 cells/mm³), which further dropped to 40,000 cells/mm³ by the next day. Due to worsening clinical status, he was referred to our hospital for further management.

On day seven of illness, the patient developed a cough and worsening shortness of breath. On arrival at our emergency room, he was hypotensive (systolic blood pressure: 80 mmHg) and hypoxic (SpO₂: 60%). A 500 mL fluid bolus was administered, and noradrenaline was continued. Oxygen supplementation was initially provided via a Venturi mask, but due to increasing respiratory distress, the patient was switched to non-invasive ventilation. Arterial blood gas analysis showed metabolic acidosis with normal lactate levels, and an electrocardiogram revealed sinus tachycardia. Cardiac biomarkers, including troponin I and creatine kinase-MB (CK-MB), were elevated. Two-dimensional (2D) echocardiogram showed a normal study. Despite initial supportive measures, the patient’s respiratory status deteriorated, necessitating endotracheal intubation and mechanical ventilation, following which he was transferred to the medical intensive care unit.

**Figure 1:**
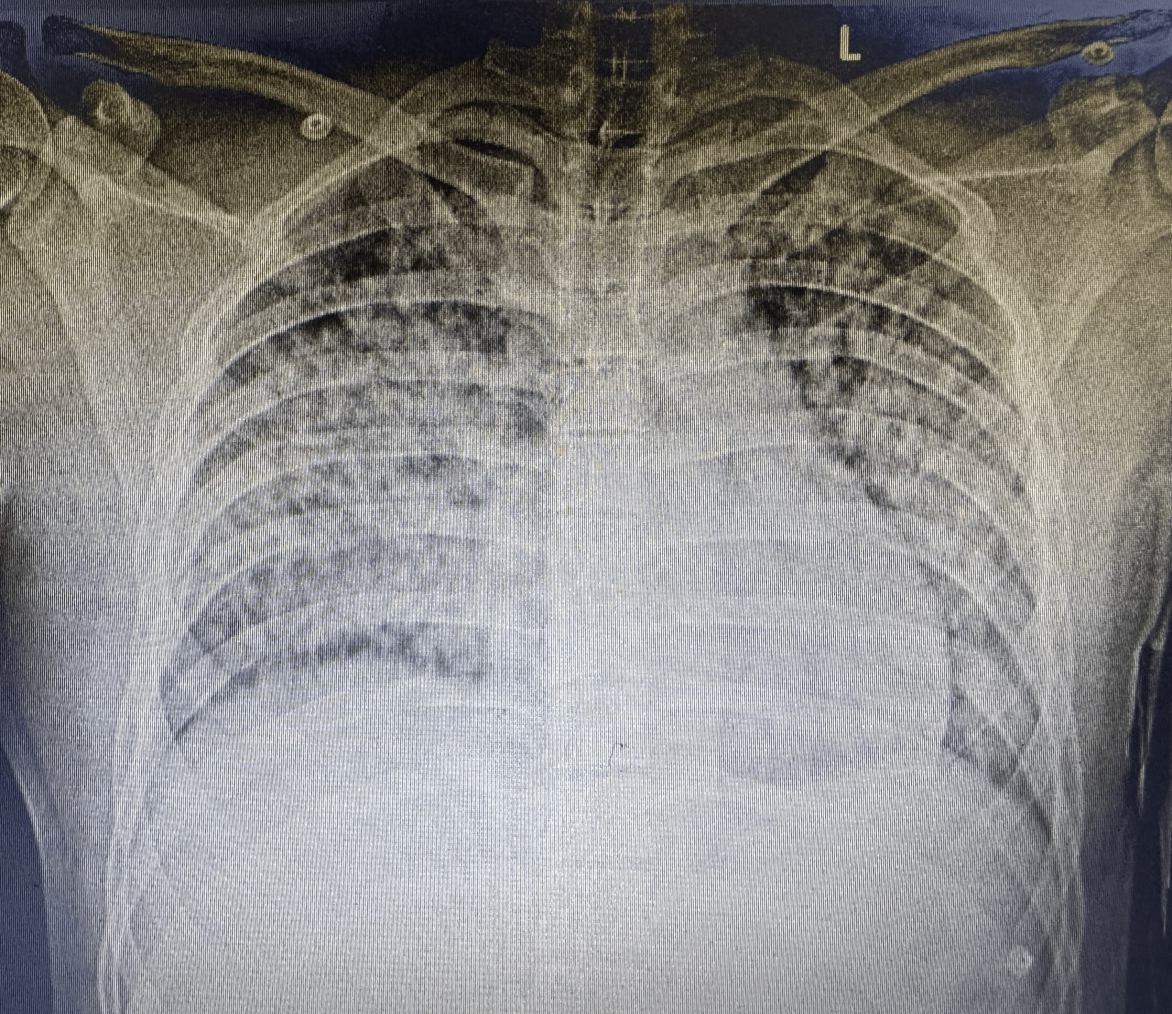
Chest X-ray showing bilateral diffuse non-homogenous fluffy opacities suggestive of diffuse pulmonary hemorrhage.

Laboratory investigations revealed hemoglobin of 8.9 g/dL, total leukocyte count of 10,990 cells/mm³, and severe thrombocytopenia with a platelet count of 12,000 cells/mm³. Liver function tests showed elevated bilirubin levels (total bilirubin: 3.26 mg/dL, direct bilirubin: 2.2 mg/dL) and elevated transaminases (serum glutamic-oxaloacetic transaminase: 172 IU/L, serum glutamic-pyruvic transaminase: 60 IU/L). Renal function was also impaired, with blood urea nitrogen of 65 mg/dL and creatinine of 3.7 mg/dL. Coagulation studies revealed an international normalized ratio of 1.08 and an elevated activated partial thromboplastin time of 62 seconds (Table 1). Chest X-ray demonstrated diffuse fluffy non-homogenous opacities, suggestive of diffuse alveolar hemorrhage, which was further confirmed by CT thorax (Figure 1). Given the presence of jaundice, thrombocytopenia, renal dysfunction, and pulmonary involvement, a preliminary diagnosis of Weil’s disease (severe leptospirosis) was made. However, dengue, vasculitis, and viral infections were considered differential diagnoses. Initial testing for *Leptospira* IgM (day seven of illness) was negative, as were serologies for dengue IgM, scrub typhus IgM, and respiratory viral panel (including influenza A/B, respiratory syncytial virus, and COVID-19). Blood and urine cultures were sterile. A bronchoscopy with bronchoalveolar lavage showed diffuse alveolar macrophages, pearl’s stain was positive, and no organisms were identified on Gram staining and multiplex polymerase chain reaction (PCR).

**Table 1:** Laboratory findings. DOI = day of illness; BUN = blood urea nitrogen; SGOT = serum glutamic-oxaloacetic transaminase; serum glutamic-pyruvic transaminase; INR = international normalized ratio; APTT = activated partial thromboplastin time

Date	Date	Date	Date	Date	Date	Date	Date	Reference range
Day significance	DOI-5	DOI-6	Admission DOI-7	Day 2 of antibiotics	Day 1 of corticosteroids	DOI-12	DOI-16	13.0–17.0 g/dL (male)
Hemoglobin (in g/dL)	-	-	8.9	8.3	10.8	11.9	11.6	13.0–17.0 g/dL (male)
Total counts (in cells/mm³)	-	-	10,990	8,160	12,470	35,320	15,510	4,000–11,000 cells/mm³
Polymorphs	-	-	79.3	76.7	71.8	79.8	65	40–75%
Platelet count (in cells/mm³)	70,000	40,000	12,000	29,000	57,000	67,000	158,000	150,000–450,000 cells/mm³
Creatinine (in mg/dL)	-	-	3.7	1.3	1.1	-	0.6	0.6–1.3 mg/dL
BUN (in mg/dL)	-	-	65	38	46	-	19	7–20 mg/dL
Direct bilirubin (in mg/dL)	-	-	2.2	-	1.94	-	0.77	0.0–0.3 mg/dL
Indirect bilirubin (in mg/dL)	-	-	1.06	-	1.92	-	0.79	0.1–1.0 mg/dL
SGOT (in IU/L)	-	-	172	-	74	-	27	0–40 IU/L
SGPT (in IU/L)	-	-	60	-	48	-	57	0–40 IU/L
INR	-	-	1.08	1.06	-		-	0.8–1.2
APTT (in seconds)	-	-	62.2	26.2	-	-	-	25–35 seconds

The patient was initially started on intravenous doxycycline and empirical broad-spectrum coverage with meropenem due to hemodynamic instability. Due to worsening respiratory status and evidence of alveolar hemorrhage, intravenous corticosteroids were initiated empirically on day nine of illness. The patient was clinically improving. Despite the negative initial serology, *Leptospira* IgM was repeated on day 11 of illness, given strong clinical suspicion, and was found to be positive. Over the next few days, the patient’s clinical condition improved. Meropenem was switched to ceftriaxone as *Leptospira* IgM was positive. Repeat cardiac markers troponin I and CK-MB were negative. The platelet count was improving. Noradrenaline was gradually tapered and discontinued, and he was extubated on day 12 of illness (five days after initiating doxycycline therapy). Oxygen supplementation was gradually weaned off, and the patient was discharged in stable condition. A *Leptospira* MAT was performed after discharge, which showed serovars *autumnalis*, *ballum*, and *hebdomadis*, confirming the diagnosis.

## Discussion

This case highlights several key aspects of leptospirosis, including its severe manifestations, diagnostic challenges, and management.

Severe manifestations of leptospirosis

Leptospirosis typically presents with mild febrile illness; however, in severe cases (Weil’s disease), multi-organ involvement occurs, including renal and hepatic dysfunction, thrombocytopenia, and pulmonary complications. One of the most severe respiratory manifestations, LPHS, can lead to acute respiratory distress syndrome and has a mortality rate of 50-70% when associated with massive hemorrhage [5,6]. DAH, as seen in our case, is a rare but life-threatening pulmonary complication of leptospirosis that requires immediate intervention with respiratory support and corticosteroids [5]. Thrombocytopenia, another key feature observed in our patient, is well-documented in severe leptospirosis. While platelet consumption due to disseminated intravascular coagulation is a possibility, studies suggest that immune-mediated platelet destruction and endothelial dysfunction contribute significantly to thrombocytopenia [8]. A platelet count below 50,000 cells/mm³ is often linked to an increased risk of hemorrhagic complications, necessitating close monitoring.

Challenges in diagnosis

Leptospirosis is often misdiagnosed due to non-specific symptoms and overlap with other tropical febrile illnesses such as dengue, scrub typhus, and malaria [3]. The delayed seroconversion seen in our case, where initial *Leptospira* IgM ELISA was negative and later turned positive, is a known diagnostic limitation. *Leptospira* IgM antibodies typically appear five to seven days after symptom onset but may remain undetectable in early illness [7]. This underscores the importance of repeat testing in clinically suspected cases. MAT is considered the gold standard for leptospirosis diagnosis, but it has several limitations, including low sensitivity in early disease, labor-intensive methodology, and subjective interpretation [4]. In endemic settings, molecular diagnostics such as PCR-based assays can be useful in early diagnosis, particularly when serology is inconclusive [3].

Use of corticosteroids in severe leptospirosis

The use of intravenous corticosteroids in severe leptospirosis, particularly cases complicated by DAH and LPHS, remains controversial. However, several studies suggest that steroids may reduce inflammation and capillary leak, potentially improving survival in critically ill patients [5]. In our case, corticosteroids were initiated due to worsening respiratory failure, leading to gradual improvement in oxygenation and extubation by day 12. Larger randomized trials are needed to establish standardized guidelines for corticosteroid use in leptospirosis.

Prognosis and follow-up

Survivors of severe leptospirosis, especially those requiring mechanical ventilation, may experience prolonged recovery. Post-leptospirosis fatigue syndrome and residual renal impairment have been reported, necessitating long-term follow-up for patients with severe disease [6]. Fortunately, our patient made a full recovery and was discharged in stable condition, highlighting the importance of early diagnosis, aggressive supportive care, and timely initiation of antibiotics in improving outcomes.

## Conclusions

This case underscores the diagnostic challenges, severe complications, and management strategies for leptospirosis with DAH with severe thrombocytopenia and cardiac involvement. In endemic areas, clinical suspicion remains paramount, even in the absence of early serological confirmation. The role of repeat serological testing and early empirical antibiotic therapy is crucial in improving outcomes. The use of corticosteroids in severe pulmonary leptospirosis warrants further investigation through large-scale clinical studies.
